# Ongoing under-reporting of clinically relevant safety data in phase II studies of tyrosine kinase inhibitors

**DOI:** 10.1038/sj.bjc.6605628

**Published:** 2010-03-30

**Authors:** P Schöffski, D H Garfield, A Hercbergs, P Wolter

**Affiliations:** 1 Department of General Medical Oncology, University Hospitals Leuven, Leuven Cancer Institute, Herestraat 49, Leuven 3000, Belgium; 2 University of Colorado Comprehensive Cancer Center, Denver, CO, USA; 3 The Cleveland Clinic, Department of Radiation Oncology, Cleveland, OH 44195, USA

**Sir**,

We read with some concern in the 13 October issue of your journal the report of the outcome of the phase II study of Novello *et al* (2009) on continuous daily sunitinib dosing in patients previously treated with platinum-based chemotherapy for advanced non-small-cell lung cancer. The trial suggests the potential clinical benefit of this multitargeted tyrosine kinase inhibitor in terms of progression arrest and shrinkage of target lesions on fashionable waterfall plots. This showed one objective partial response among 47 patients (2.1%) treated with a continuous dosing of sunitinib. The interpretation of the efficacy signal generated by this trial is hampered by the non-randomised, non-controlled design of the study. The objective response rate did not meet the pre-defined criterion required to reject the null hypothesis, as at least five objective responses would have been required for a positive outcome of the trial.

A considerable number of patients required dose and schedule modifications. Globally, one out of four patients (25%) discontinued treatment due to adverse events, including four who stopped treatment during cycle 1 for side effects, once again illustrating that multi-targeted tyrosine kinase inhibitors should not be considered as an easy-to-administer, convenient alternative to classical chemotherapy, especially not in a pretreated, often-elderly cohort of patients with highly refractory solid tumours.

The assessment of safety was defined as a secondary end point in this trial, but the type and frequency of laboratory tests for biochemical evaluation of safety performed during the conduct of the trial were not specified by the authors. We note with surprise that patients participating in the study were not routinely screened either for sunitinib-induced thyroid dysfunction or for cardiac toxicity. There is no mention of routine thyroid or cardiac function assessment during sunitinib treatment. In contrast to this, hypothyroidism, first reported by Desai *et al* (2005), is a well-known adverse side effect of sunitinib (Wolter *et al*, 2008a; Torino *et al*, 2009). Remarkably, the authors report symptoms that are possibly attributable to hypothyroidism in a large proportion of their patients, including a 59.6% incidence of treatment-emergent fatigue (17% of the cases that were categorised as grade 3/4 events), but we could not find any information as to whether this was related to thyroid dysfunction. This is a significant issue because such symptoms might be due to sunitinib-induced hypothyroidism and could therefore be reversible with thyroid hormone replacement. Secondly, fatigue can also be related to cardiac failure, which also has recently been reported as an important side effect of sunitinib (Chu *et al*, 2007; Schmidinger *et al*, 2008; Telli *et al*, 2008; DiLorenzo *et al*, 2009). Unfortunately, detailed information on cardiac toxicity is also lacking, but the authors reported that at least one patient died because of treatment-related congestive heart failure.

According to a growing number of publications, if systematically assessed by thyroid hormone determination, thyroid dysfunction is seen in 30–60% of patients treated with sunitinib. In some cases thyroid damage can even be irreversible, leading to the need for long-term thyroid hormone replacement therapy (Wolter *et al*, 2008a; Torino *et al*, 2009; Rogiers *et al*, 2010), which, however, should be carefully individualised (Garfield *et al*, 2007) in light of the evidence that L-thyroxine has been shown to be a growth factor in solid cancers acting via a non-genomic mechanism (i.e., through the av beta 3 integrin receptor to activate tumour cell proliferation; Lin *et al*, 2009). The determination of TSH levels in all patients treated with sunitinib at baseline and during treatment, both within and outside of clinical trials, is recommended by most experts in the field (Kollmansberger *et al*, 2007; Bhojani *et al*, 2008). Recently, we have proposed an algorithm in this journal to deal with this problem in daily clinical practice (Wolter *et al*, 2008a). Indeed, Hellevik *et al* (2009) have reported that in a large prospective population (*n*=30 000) study of blood thyroid hormone levels in individuals without a prior diagnosis of cancer, a low TSH level was associated with an increased risk for developing lung and prostate cancer – a risk factor that increases with time.

Furthermore, there are preliminary data showing that thyroid dysfunction under sunitinib treatment might be a surrogate marker for clinical outcome (Wolter *et al*, 2008b). In a prospective study of sunitinib-induced thyroid dysfunction in patients with advanced renal cell carcinoma, the median progression-free survival of patients with thyroid abnormalities was 10.3 months, while for those without the abnormalities it was 3.6 months (*P*=0.047, log rank test). In addition, the group with thyroid dysfunction had a median overall survival of 18.2 months compared with 6.6 months in the euthyroid group (*P*=0.13) (Wolter *et al*, 2008b). To further explore thyroid dysfunction as a possible surrogate marker for efficacy, the determination of TSH might have been important in the above-mentioned study.

In summary, we cannot conclude that the secondary end point of the present study, namely, to assess the safety of sunitinib in this patient population, was entirely met, as at least two important and well-known side effects were not routinely assessed. We recommend that the safety assessment in patients treated with sunitinib and other tyrosine kinase inhibitors should always include thyroid and cardiac function monitoring to avoid underreporting of adverse events, to adequately manage potentially reversible side effects and to help identify off-target drug effects as a possible surrogate marker for efficacy.
